# Evaluation of parents' knowledge, attitudes, and practices regarding self-medication for their children’s dental problems during the COVID-19 pandemic: a cross-sectional survey

**DOI:** 10.1186/s12903-021-01466-7

**Published:** 2021-03-05

**Authors:** Emine Sen Tunc, Emre Aksoy, Hatice Nilden Arslan, Zeynep Kaya

**Affiliations:** 1 Department of Pediatric Dentistry, Faculty of Dentistry, Ondokuz Mayis University, 55270 Samsun, Turkey; 2 Department of Public Health, Faculty of Medicine, Ondokuz Mayis University, Samsun, Turkey

**Keywords:** COVID-19, Pediatric dentistry, Self-medication

## Abstract

**Background:**

Self-medication refers to taking medicine without consultation with a doctor or dentist, and it is an important health issue, especially during the COVID-19 pandemic. There are no data about parents’ SM practices for their children’s dental problems during the COVID-19 pandemic. The present study aims to evaluate parents’ knowledge, attitudes, and practices regarding self-medication for their children’s dental problems during the COVID-19 pandemic in Northern Turkey.

**Methods:**

A cross-sectional survey was carried out in the pediatric dental clinic at Ondokuz Mayis University, Faculty of Dentistry, Department of Pediatric Dentistry, immediately after the COVID-19 lockdown ended. A total of 389 parents who agreed to participate in the study completed the questionnaire from July 1 to October 1. A questionnaire with 18 items was designed to collect information on the parents’ knowledge and attitudes regarding when, why, and how to use drugs and on their practices on medicating their children. The collected data were analyzed using descriptive and analytical statistics (chi-square test).

**Results:**

The majority of parents (n = 273; 70.2%) practiced self-medication for their children's dental problems. Self-medication with a previously prescribed medications was usually preferred by parents (n = 179; 62.2%). Analgesics (98%) were the most commonly used medicines by parents in their self-medication for their children's dental problems.

**Conclusion:**

Prevalence of self-medication practices for children's dental problems is high in Turkey during the COVID-19 pandemic. Therefore, new healthcare services, such as teledentistry, may be useful to overcome problems related to the self-medication of children during times when the ability to reach healthcare providers is limited, such as during pandemics.

**Supplementary Information:**

The online version contains supplementary material available at 10.1186/s12903-021-01466-7.

## Background

COVID-19 (coronavirus disease 2019) is the disease caused by a novel coronavirus. SARS-CoV-2 (originally identified as 2019-nCoV) infection can cause many systems, such as cardiovascular, endocrine, and gastrointestinal systems, but particularly affects the respiratory system, and it can be life-threatening. As of March 11, 2020, a pandemic was declared by the WHO (World Health Organization) as a consequence of the spread of the infection to many countries in a few months [1, 2]. The first COVID-19 case in Turkey was reported on March 10, 2020 [3]. Several measures were taken, particularly in the health sector, so that the public health system in Turkey would not become overloaded. Elective procedures and nonurgent dental treatments were delayed or rescheduled, and only emergency treatments and procedures were accepted by health care providers. Severe toothaches, abscesses with systemic complaints, dental trauma, and life-threatening or uncontrolled bleeding were defined as oral emergencies [4, 5].

The Turkish Ministry of Health announced the completion of the first phase of the COVID-19 control phase and the initiation of the second phase, termed the controlled social life phase. Although this phase was declared in early May, strict measures were enforced until the end of May. As of June 1, standard infection prevention protocols were implemented in compliance with the guidelines issued by the Turkish Ministry of Health, and all dental operations, both urgent and nonurgent, began to be carried out. During the lockdown and the restriction of dental work, patients had trouble accessing dental care providers, and several elective procedures for dental care providers had to be postponed. During this time, patients were assumed to more commonly practice self-medication for dental problems [3–5].

Self-medication (SM) is defined as “the taking of drugs, herbs or home remedies on one's own initiative, or on the advice of another person, without consulting a doctor” [6]. SM patterns are influenced by many factors, such as a lack of knowledge regarding the side effects of medicines, socioeconomic status, difficulties accessing health services, epidemic diseases, and patients’ beliefs and norms [7]. Patients may use medicines based on their previous experiences, buy medicines based on a close relative’s advice, use the same medicines they have previously used, or buy medicines directly from the pharmacy without seeing a doctor or dentist. Thus, a dangerous habit of abuse has emerged through SM without consulting a doctor, either independently or with advice from someone else, which is the result of a medical model that people have learned [8, 9].

There are two kinds of nonprescription medicines defined based on their accessibility. These are over-the-counter drugs (OTCs) and behind-the-counter drugs (BTCs). OTCs are used to relieve simple ailments in daily life and are sold directly to patients without them seeing a healthcare professional. OTCs include analgesics, vitamin and mineral supplements, antacids, and decongestants. BTCs are an intermediate category of medicines between OTCs and prescription medicines. Unlike OTCs, BTCs have some restrictions, such as the need to show an ID card to purchase them. The status of some medicines, such as antibiotics and narcotic analgesics, prevents their direct sale to patients without a doctor’s or dentist’s prescription [10, 11]. Turkey has legislation requiring that medicines such as antibiotics and narcotic analgesics are dispensed with a prescription [12]. Although antibiotics are not available without a prescription, many physicians and dentists prescribe these medicines on demand. Patients have these medicines prescribed by their physician and store them for later use at home. Additionally, antibiotics are available without a prescription on many illegal websites around the world. As the unnecessary use of antibiotics increases the risk of developing antibiotic resistance, this situation poses a very serious danger [13, 14].

Internet search trends for SM were shown to be increasing as a result of restricted patient admissions in hospitals during COVID-19 and concerns about contracting COVID-19 [15, 16]. The prevalence of SM varies, especially in terms of a higher prevalence in developing countries [7–9, 17, 18], but there is little knowledge about SM during the pandemic period. Additionally, there is a lack of studies on the SM practices of parents for their children with dental problems. The present study aims to evaluate parental knowledge, attitudes, and practices about SM for their children among parents who applied to the public pediatric dental clinic regarding their children’s dental problems during the COVID-19 pandemic in Northern Turkey.

## Methods

### Study area

A cross-sectional survey was carried out from July 1 to October 1 during the controlled social life phase with the approval of the Ethical Boards and Commissions of Ondokuz Mayis University, Samsun, Turkey (2020/461).

Ondokuz Mayis University Faculty of Dentistry, which began service in 1994 in Samsun, is located in the largest and most populous city in Turkey's Black Sea region. The population of the city of Samsun is approximately 1.5 million. Patients from other neighboring provinces in the Black Sea region also visit the university’s clinic. Prior to the COVID-19 pandemic, approximately 60–80 patients applied to this pediatric dental clinic daily. The number of daily patient applications was restricted to 20 because of the safety measure to minimize the transmission that had to be taken due to the COVID-19 pandemic.

### Data collection

A convenience sampling method was employed. Questionnaires were administered to 396 parents when they arrived for a dental visit at the public pediatric dental clinic. A total of 389 parents agreed to participate, and only 7 of 396 parents declined to participate in this study (response rate of 98.2%).

*Inclusion criteria* Parents with children between 0–12 years of age who applied to the pediatric dental clinic and who agreed to participate in the study.

*Exclusion criteria* Parents who did not agree to participate.

This study followed established guidelines for reporting medical surveys [19]. The researchers designed the questionnaire by adapting questions from previous surveys [20, 21]. A prequestionnaire was administered to 10 patients of similar ages with the aim of detecting comprehension problems and to assess if the questions responded to the research aims. After the prequestionnaire, a few questions were modified, and the implementation phase started. Data were collected in a face to face interview on the revised questionnaire. Sociodemographic data such as parents’ age, gender, level of education, economic status, health insurance and children’s age, gender, and medical history were also collected during this research.

The revised questionnaire with 18 items was used to gather the opinions of parents regarding SM for their children’s dental problems as follows: the item on the frequency of SM for their children’s dental problems was collected with two response options, yes or no. Other items were collected with multiple-choice questions; these items assessed the dental disease types for which SM was practiced; groups of medicines used by parents for their children, such as antibiotics and analgesics; types of sources of medical data; ways of obtaining medicines; knowledge of adverse reactions for medicines; time frame of medicine use; and reasons for SM. The survey questions can be found in Additional file 1: Appendix 1.

### Statistical analysis

The data were statistically analyzed using SPSS software version 25 (IBM Corp, Armonk, NY, USA). Descriptive analyses were performed for demographic data. Chi-square test was used for testing the statistically significant differences between variables. The statistical significance was set at *p* ≤ 0.05.

## Results

Characteristics of the study population and factors associated with self-medication practices are presented in Table 1.

**Table 1:** Characteristics of the study population and factors associated with practices of self-medication, n (%)

	Total (n = 389)	Self-medication present*		*p*
		Yes	No	
		(n = 273)	(n = 116)	
*Children gender*				
Boy	202 (51.9)	150 (74.3)	52 (25.7)	.068
Girl	187 (48.1)	123 (65.8)	64 (34.2)	
*Age*				
0–4	36 (9.3)	25 (69.4)	11 (30.6)	.97
5–8	185 (47.6)	129 (69.7)	56 (30.3)	
9–12	168 (43.2)	119 (70.8)	49 (29.2)	
*Parents gender*				
Male	104 (26.7)	69 (66.3)	35 (33.7)	.318
Female	285 (73.3)	204 (71.6)	81 (28.4)	
*Age*				
25–30	46 (11.8)	32 (69.6)	14 (30.4)	.773
30–35	97 (24.9)	72 (74.2)	25 (25.8)	
35–40	144 (37.1)	100 (69.4)	44 (30.6)	
> 40	102 (26.2)	69 (67.6)	33 (32.4)	
*Educational level*				
Basic education	102 (26.2)	65 (63.7)	37 (36.3)	.224
Secondary education	163 (41.9)	120 (73.6)	43 (26.4)	
Higher education	124 (31.9)	88 (71)	36 (29)	
*Urbanicity*				
Rural	42 (10.8)	33 (78.6)	9 (21.4)	.208
Urban	347 (89.2)	240 (69.2)	107 (28.4)	
*Economic status*				
Lower income	81 (20.8)	51 (70.8)	21 (29.2)	.572
Middle income	236 (60.7)	169 (71.6)	67 (28.4)	
High income	72 (18.5)	53 (65.4)	28 (34.6)	
*Health insurances*				
Present	358 (92.0)	251 (70.1)	107(29.9)	.92
Absent	31 (8.0)	22 (71.0)	9 (29.0)	

A total of 389 participants completed the questionnaire during the study period. The majority of the parents (n = 287; 73.8%) were under the age of 40 years. Additionally, most parents (n = 287; 73.8%) had at least a secondary education degree. A small proportion of participants (n = 42; 10.8%) lived in rural areas.

The review of the children’s medical histories showed that a small proportion of children (n = 39; 10%) had chronic diseases; a large proportion (n = 32; 82%) of these children with chronic diseases constantly used medication. The proportion of parents’ who self-medicated their children was lower among basic education graduates (63.7%) than among parents with other educational levels, but no significant difference in self-medication was found by parental educational level (*p* > 0.05).

**Fig. 1:**
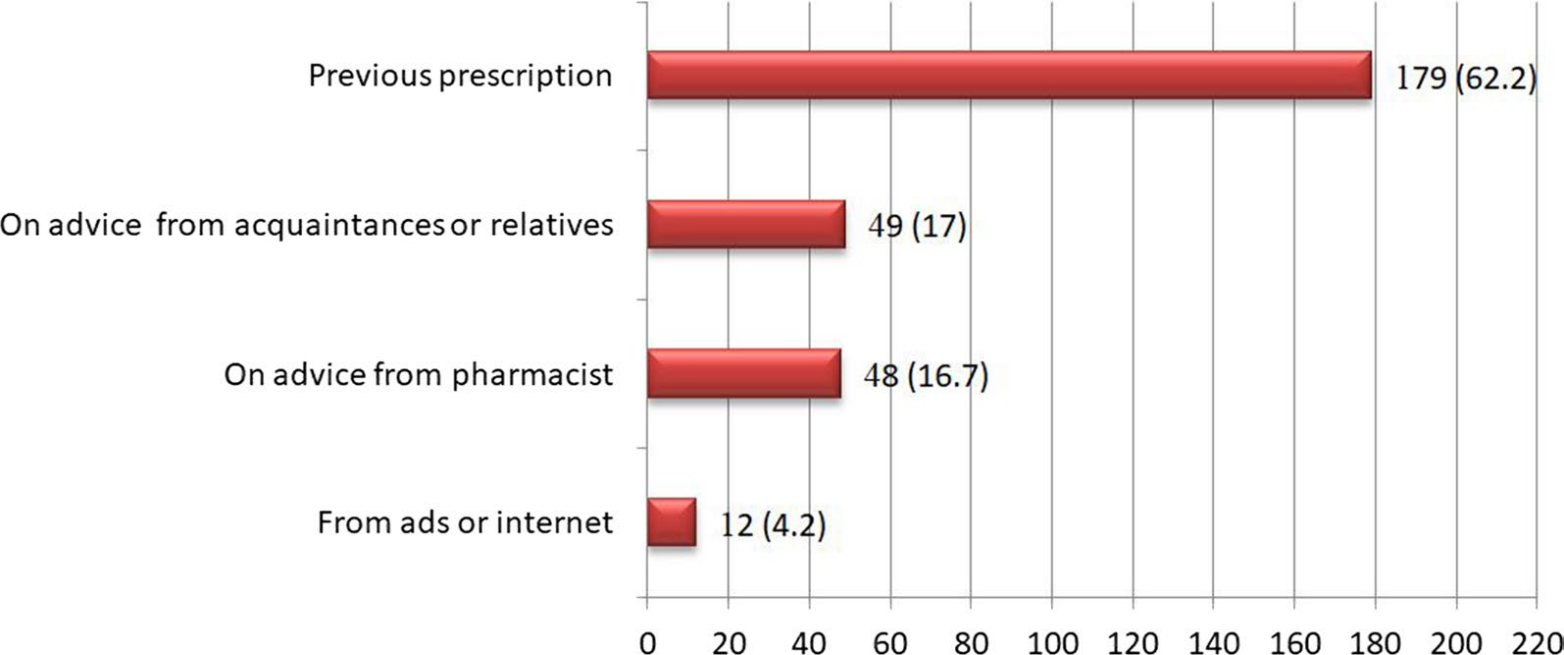
Number of parents that resort to certain solution when their children's dental problems occur, n (%)

When their children had dental problems, a high percentage (n = 272; 70%) of parents self-medicated their children. Of the parents who practiced SM using previous prescriptions (n = 179; 62.2%), only 12 (4.2%) of the parents practiced any medicines from advertising (ads) or the internet (Fig. 1). Some parents practiced SM of their children with analgesics (n = 268; 98%), antibiotics (n = 104; 38.1%), mouthwashes (n = 36; 13.1%) and herbal medicines (n = 24; 8.8%). Additionally, analgesics were the most preferred medicine for toothaches (n = 180; 83.3%) (Table 2).

**Table 2:** Participants’ beliefs about requirements for a medicine for their children’s dental problems, (n = 273)*

Dental Problems	Analgesics n (%)	Antibiotics n (%)	Mouthwash n (%)	Herbal Medicine n (%)
Toothache	180 (83.3)	62 (28.7)	21 (9.7)	13 (6.0)
Tooth abscess	26 (74.3)	20 (57.1)	5 (14.3)	1 (2.9)
Facial swelling	11 (84.6)	10 (76.9)	3 (23.1)	1 (7.7)
Eruption disturbances	7 (63.6)	1 (9.1)	0 (0)	2 (18.2)
Gum problems	7 (58.3)	3 (25)	6 (50)	2 (16.7)
Dental trauma	7 (100)	2 (28.6)	1 (14.3)	0 (0)
Other problems (Bruxism, TMJ problems, Tooth colored problems)	30 (73.2)	6 (14.6)	0 (0)	5 (12.2)
Total	268 (98.0)	104 (38.1)	36 (13.2)	24 (8.8)

^*^Row percentages used in this table, and more than one option was chosen in some questions

Some parents (n = 140; 45.6%) thought that after symptoms disappeared, medication could be stopped, some (n = 159; 51.8%) thought that treatment should be continued as recommended by the doctor or dentist, and some (n = 11; 36.2%) thought that treatment could be stopped when the medicines ran out.

**Fig. 2:**
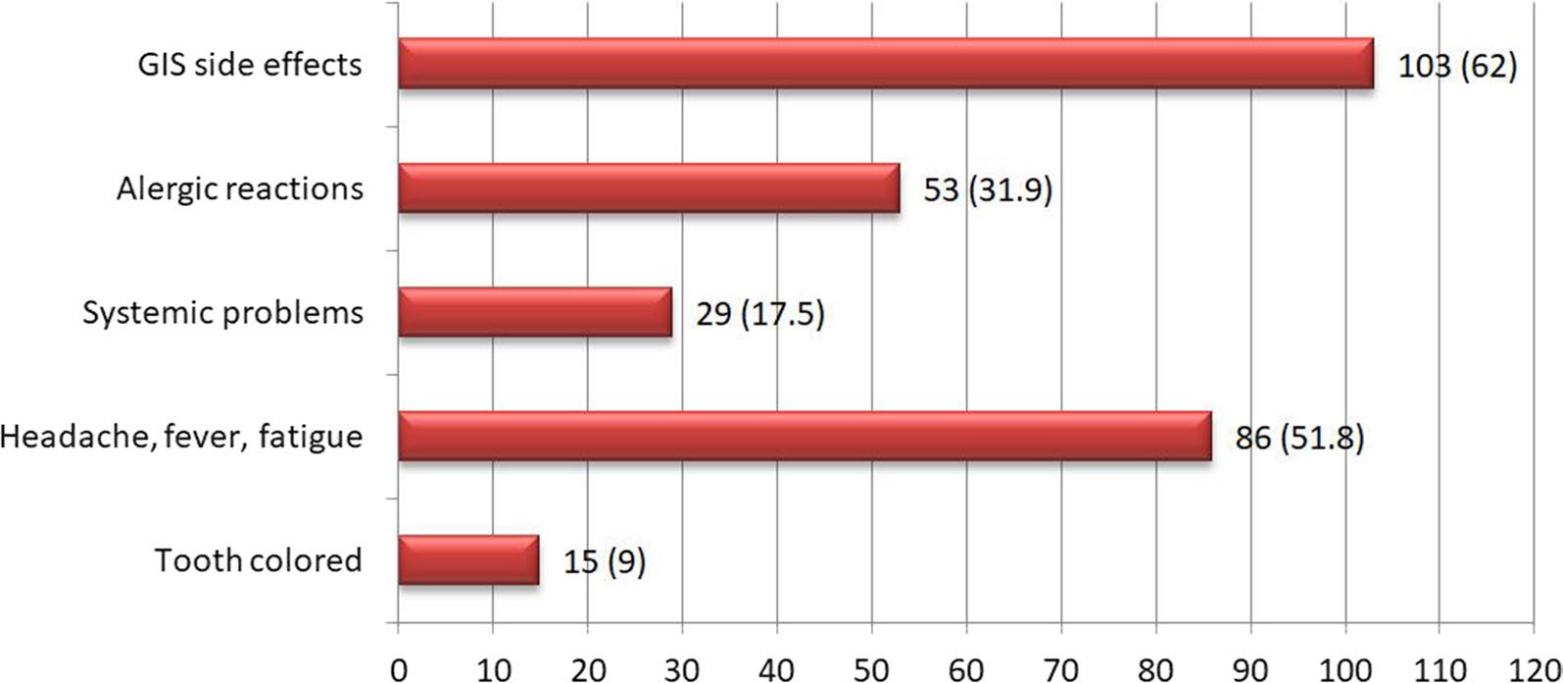
Number of parents who know side effects when practicing self-medication, n (%)

Less than half of the parents (n = 166; 42.7%) had limited knowledge of side effects. Of these parents, 103 (62%) thought that the medicines had side effects on the gastrointestinal system (GIS) (Fig. 2).

**Fig. 3:**
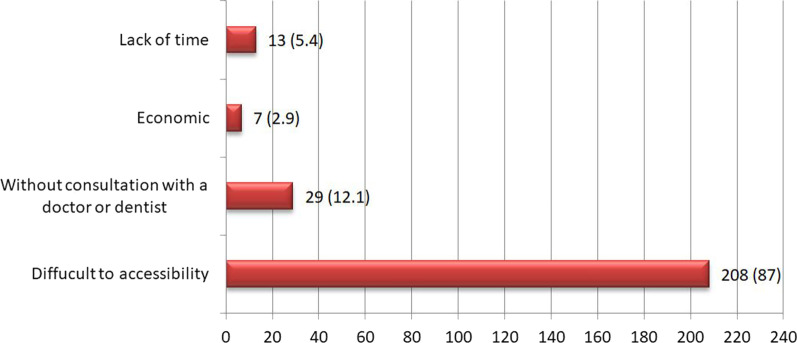
Reason for practicing self-medication, n (%)

As reported by the parents who practiced SM (n = 208; 87%), the main reason for self-medication was difficulty obtaining a dental consultation (Fig. 3).

## Discussion

Although there are many studies about SM practices among adult patients, there is a lack of studies on SM practices for children with dental problems. In this study, an extremely high prevalence (70.2%) of SM was found in children with dental problems in Northern Turkey. The prevalence of SM practices for children was previously found to be 70% in the Romanian population [20], 58% in Pakistan [22], 56.6% in Brazil [9], 53.8% in Turkey [23], 45% in Denmark [24], and 25.2% in Germany [8]. This study result agrees with that reported by Tarciuc et al. [20], but the observed prevalence is higher than that reported in other studies [8, 9, 22, 24]. In addition, our result is higher than Yazici et al.’s study that evaluated parental self-medication in children with upper respiratory tract infection in Turkey [23]. This result can be attributed to the increasing trend of SM behavior in the COVID-19 pandemic.

The prevalence of SM depends on several factors, such as education level, socioeconomic status, and accessibility to the health system [7]. With COVID-19, access to healthcare providers has become very difficult for patients, which is thought to further increase the practice of SM, which is known as an important health issue worldwide [15]. A recent study of Google interest trends for self-medication during the COVID-19 pandemic showed an increase in the number of self-medication searches worldwide since the COVID-19 pandemic, which indicates higher interest in self-medication around the world [15]. This finding shows that it is necessary to reach people in different ways, such as through teledentistry. Over the past decade, teletechnology, a fast, simple and reliable way of reaching patients, has attracted more attention in both medicine and dentistry. Teledentistry is intended to enhance patients' dental care and time management. It offers access patients to a dentist, reduces waiting lists and reduces consultation time [25].

In the current study, mothers (73.3%) were generally the parents who accompanied the children. This finding clearly shows that women are more likely than fathers to follow up with medical and dental appointments regarding the health of their children and is similar to the results of other studies [20, 22, 24, 26–28].

In the present study, no significant statistical results were found between self-medication administration and the demographic characteristics of the participating families. Although our results are in line with those of Jensen et al. [24], some studies found that demographic characteristics were significantly related to the practice of SM [8, 20, 26]. Surprisingly, these studies found that the prevalence of SM was higher in highly educated parents [8, 20, 26]. Additionally, Du Y et al. reported that self-medication administration was positively associated with parents’ socioeconomic status [8].

The vast majority of parents (67.7%) in the current study reported that they practiced SM using medicines previously prescribed to their children, and the observed proportion is higher than that reported in other studies [8, 20, 26, 28]. Approximately the same proportion of parents (17%) reported that they practiced SM based on advice from close relatives and pharmacists. This finding can be explained by difficulties in accessing healthcare providers during COVID-19.

Many studies have found that analgesics are the most commonly used medicines in SM for children [9, 20, 22], which is consistent with the findings of this study. The common use of analgesics may be associated with their availability and low price. In addition, parents may believe that they are not toxic or can do little harm.

In the current study, toothache was the major trigger for parents practicing SM of their children. Studies have shown that the major trigger of SM in adults is toothache [21, 29]. In this study, less than half of the parents (42.7%) had limited knowledge of side effects, which may be because the parents did not have sufficient information on the side effects of medicines. This finding was previously reported by another study on parents practicing SM for their children [20]. The current study also showed that most parents who practiced self-medication thought it was difficult to access health care providers during COVID-19.

## Limitation

The present study has some limitations associated with the characteristics of survey studies. The survey was conducted during the pandemic period after the lockdown ended. The prevalence of SM was assumed to be higher than that before the pandemic because more children with urgent dental problems came to the clinic in this period.

## Conclusion

Prevalence of self-medication practices for children's dental problems is high in Turkey during the COVID-19 pandemic.

Accessing patients in their homes can prevent self-medication during public crises that make it difficult for patients to reach healthcare providers, such as pandemics; thus, new healthcare approaches, such as teledentistry, are needed.

## Supplementary Information

**Additional file 1**. Appendix 1–Survey questions: Parent questionnaire regarding self-medication for him/ her child's dental problems.

## Data Availability

All data generated or analyzed during this study are included in this published article (and its supplementary information files).

## Abbreviations

BTCs: Behind-the-counter drugs
GIS: Gastrointestinal system
ID: Identity document
SM: Self-medication
ads: Advertising
TMJ: Temporomandibular joint
OTCs: Over-the-counter drugs
