# Supplementary material for: Evaluation of parents' knowledge, attitudes, and practices regarding self-medication for their children’s dental problems during the COVID-19 pandemic: a cross-sectional survey
Source: BMC Oral Health. 2021 Mar 5;21:98. doi: 10.1186/s12903-021-01466-7 (PMC7934979; doi:10.1186/s12903-021-01466-7)
Supplement: Supplementary file 1 — Additional file 1. Appendix 1–Survey questions: Parent questionnaire regarding self-medication for him/ her child's dental problems. [file 12903_2021_1466_MOESM1_ESM.docx]

Appendix 1 – Survey questions

1. Child’s age?

☐0-4 years

☐5-8 years

☐9-12 years

1. Child’s gender?

☐Girl

☐Boy

1. Parent’s age?

☐25-30 years

☐30-35 years

☐35-40 years

☐Over 40 years

1. Parent’s gender?

☐Woman

☐Man

1. What is the level or degree of your education?

☐Primary school

☐Secondary school

☐High school

☐University

☐Residency or doctorate

1. Where do you live?

☐City

☐Country

☐Village

1. What is your income?

☐ Income is more than expenses

☐ Income is balanced to expenses

☐ Expenses is more than income

1. Do you have any health insurance?

☐Yes

☐No

1. Any medical conditions that your child has? If yes, please clarify.

☐Yes …………………………………………………

☐No

1. Any regular medications your child require?

☐Yes

☐No

1. Have you ever treated your child (give medicine without seeing a doctor or dentist) during the COVID-19 pandemic? If yes, please clarify.

☐Yes …………………………………………………

☐No

1. If your answer to the previous question is yes, how did you obtain the medicine you used?

☐Purchase with prescription

☐Purchase from pharmacy without prescription

☐ I used medicine that is available at home

☐ I used the medicine that my relative used for her own child.

☐ I called the doctor or dentist who previously treated my child and received information about the drug that I was supposed to use.

☐Other .....................................

1. In which of the following dental-related situations do you give medicine to your child without no seeing a dentist? (Check more than one if applicable.)

☐ Toothache

☐ Tooth abscess

☐ Facial swelling

☐ Eruption disturbances

☐ Gum problems

☐ Dental trauma

☐ Bruxism

☐ TMJ problems

☐ Tooth-colored problems

1. Which of the following drug groups do you use for your child's dental problems? If you know the trade name of the drug you use, write please. (Check more than one if applicable.)

☐ Analgesic- Antipyretic……………………………………………….

☐ Antibiotic ……………………………………………….

☐Mouth wash ……………………………………………….

☐ Dental gels

………………………………………………..

☐ Herbal medicine

………………………………………………..

1. What is(are) the common adverse reaction(s) of drugs? (check more than one if applicable)

☐ Diarrhea

☐ Nausea-vomiting

☐ Stomach pain

☐ Allergic reactions

☐ Headache

☐ Fever

☐ Cruelty

☐ Kidney problems

☐ Liver problems

☐ Tooth discoloration

☐ Joint-muscle pain

☐ Sleep problems

☐ Numbness - tingling

1. Your selection of medicines for self-medication for your child was based on...?

☐Previous prescription

☐On advice from acquaintances or relatives

☐On advice from pharmacist

☐From advertising or internet

1. When did you normally stop giving medicine to your child? (check more than one if applicable)

☐ After symptoms disappeared

☐ After abscess disappeared

☐ Based on instructions

☐ If I give antibiotics to my child after antibiotic run out

☐ A few days after recovery

☐ Based on the previous experiences (Previous a doctor or dentist consultations)

☐ After three days regardless of the outcome

1. What was (were) your reason(s) for self-medication for your child in dental problems (check more than one if applicable)

☐Lack of time

☐Cost-saving

☐No doctor’s or dentist’s consultation needed

☐Difficult to accessibility

☐The COVID-19 pandemic
